# Home-Based Exercise Program for Patients With Combined Advanced Chronic Cardiac and Pulmonary Diseases: Exploratory Study

**DOI:** 10.2196/28634

**Published:** 2021-11-09

**Authors:** Cyrille Herkert, Lidwien Graat-Verboom, Judith Gilsing-Fernhout, Manon Schols, Hareld Marijn Clemens Kemps

**Affiliations:** 1 Department of Cardiology Máxima Medical Center Eindhoven Netherlands; 2 Department of Pulmonology Máxima Medical Center Eindhoven Netherlands; 3 ParaMáx: Center for Paramedic Care Máxima Medical Center Eindhoven Netherlands; 4 Department of Industrial Design Eindhoven University of Technology Eindhoven Netherlands

**Keywords:** home-based exercise, cardiac diseases, pulmonary diseases, comorbidities, elderly

## Abstract

**Background:**

As chronic cardiac and pulmonary diseases often coexist, there is a need for combined physical home-based rehabilitation programs, specifically addressing older patients with advanced disease stages.

**Objective:**

The primary aim of this study is to evaluate the completion and adherence rates of an 8-week, home-based exercise program for patients with advanced cardiopulmonary disease. The secondary end points include patient satisfaction; adverse events; and program efficacy in terms of change in functional capacity, level of dyspnea, and health-related quality of life.

**Methods:**

The participants received a goal-oriented, home-based exercise program, and they used a wrist-worn activity tracker to record their exercise sessions. Activity tracker data were made visible on a digital platform, which was also equipped with several other features such as short instruction videos on how to perform specific exercises. The participants received weekly coaching by a physiotherapist and an occupational therapist through video communication.

**Results:**

In all, 10 patients with advanced combined cardiopulmonary disease participated (median age 71, IQR 63-75 years), and 50% (5/10) were men. Of the 10 participants, 9 (90%) completed the 8-week program. Median adherence to the exercise prescription was 75% (IQR 37%-88%), but it declined significantly when the program was divided into 2-week periods (first 2 weeks: 86%, IQR 51%-100%, and final 2 weeks: 57%, IQR 8%-75%; *P*=.03). The participants were highly satisfied with the program (Client Satisfaction Questionnaire: median score 29, IQR 26-32, and Purpose-Designed Questionnaire: median score 103, IQR 92-108); however, of the 9 participants, 4 (44%) experienced technical issues. The Patient-Specific Complaints Instrument scores declined, indicating functional improvement (from median 7.5, IQR 6.1-8.9, to median 5.7, IQR 3.8-6.7; *P*=.01). Other program efficacy metrics showed a trend toward improvement.

**Conclusions:**

Home-based cardiopulmonary telerehabilitation for patients with severe combined cardiopulmonary disease is feasible in terms of high completion and satisfaction rates. Nevertheless, a decrease in adherence during the program was observed, and some of the participants reported difficulties with the technology, indicating the importance of the integration of behavior change techniques, using appropriate technology.

**Trial Registration:**

Netherlands Trial Register NL9182; https://www.trialregister.nl/trial/9182

## Introduction

### Background

Chronic cardiac and pulmonary diseases often coexist because they share similar risk factors such as older age, cigarette smoking, sedentary lifestyle, and persistent low-grade systemic inflammation [1]. The prevalence of cardiac diseases such as ischemic heart disease, chronic heart failure (CHF), and atrial fibrillation is higher in patients with chronic obstructive pulmonary disease (COPD) than in the general population (30%, 20%, and 13%, respectively) [1,2]. In addition, in patients with CHF and ischemic heart disease, COPD and asthma are more prevalent compared with patients without cardiovascular diseases [3]. As chronic cardiac and pulmonary diseases frequently overlap in symptoms and clinical course and share a high morbidity, with recurrent hospital admissions, there is a need for an integrated management of these often older and frail patients. Ideally, this applies not only to diagnostic and therapeutic regimens but also to physical rehabilitation.

Exercise-based cardiac rehabilitation (CR) and pulmonary rehabilitation (PR) are highly recommended for a wide range of cardiac and respiratory disorders because they result in improvement of exercise capacity and quality of life (QoL) and reduction in hospital admissions related to the disease [4,5]. Despite these positive effects as well as international guidelines recommending CR and PR, participation remains low. Concerning CR, approximately 30% of all eligible patients attend a CR program [6], with the lowest uptake in patients with chronic cardiovascular diseases such as CHF [7]. This underuse is attributed not only to low referral rates but also to patient-related factors such as older age, comorbidities, and larger distance to the nearest rehabilitation center [8]. As in the case of CR, PR uptake in patients with COPD is low [9]. Lack of transport, timing of the program, and burden of illness are major factors impeding PR attendance [10]. To overcome these barriers, there is an increasing interest in home-based rehabilitation programs. Home-based CR has been shown to have effects comparable with those of center-based CR on exercise capacity, health-related QoL (HRQoL), and mortality in the short term, both in patients diagnosed with coronary artery disease and in patients with CHF, with a higher compliance in home-based CR [11]. The results of a Cochrane review on telerehabilitation in patients with chronic respiratory diseases showed that there is a limited number of studies available that provide evidence for a similar outcome compared with traditional PR [12].

To date, there are 2 major gaps in evidence with respect to cardiac and pulmonary telerehabilitation. First, the studies on cardiac and pulmonary telerehabilitation mainly focus on patients classified as low risk, that is, younger patients, without major comorbidities, in a stable phase of the disease, rather than on the merging group of older and frail patients. These vulnerable patients, however, might benefit most from rehabilitation because of impairment in multiple domains of physical functioning, which lead to recurrent hospital admissions [13]. In fact, a pilot study on rehabilitation in older patients with acute decompensated heart failure showed promising results in improving physical function and reducing rehospitalization [13]. Although study retention and adherence were acceptable in this center-based rehabilitation program, offering home-based programs for these patients might be even more successful to overcome the aforementioned barriers to center-based rehabilitation, which are particularly common in this patient category. Second, cardiac and pulmonary disease management are seldom integrated. To our knowledge, there is only one trial focusing on telerehabilitation of patients with combined CHF and COPD, showing that their combined telerehabilitation program is feasible, safe, and effective [14]. As this program focused on patients with middle-severe combined CHF and COPD, the effectivity in patients with advanced combined cardiopulmonary disease remains to be established.

### Objective

To address the current gaps in telerehabilitation, this exploratory study was designed to assess the feasibility of a personalized, home-based, goal-oriented exercise program in patients with advanced, combined chronic cardiac and pulmonary diseases. The results of this study will be used to design a future, large clinical trial.

## Methods

### Study Design and Population

This is a single-center feasibility study designed to explore program completion and adherence, as well as possible barriers to adherence, of a novel, personalized, home-based telerehabilitation program for patients with combined advanced chronic cardiac and pulmonary diseases. The secondary end points include patient satisfaction; adverse events; and an exploratory analysis of program efficacy in terms of functional capacity, level of dyspnea, and HRQoL. The results of this study will be used to guide the design of a larger clinical trial. The study was performed at the Máxima Medical Center in Veldhoven, Netherlands. In all, 10 patients diagnosed with a combination of chronic cardiac and pulmonary diseases were included. The eligible patients were randomly selected from a group of patients who were participating in a telemonitoring program for combined chronic cardiac and pulmonary disease because of 1 or more hospital admissions within the past year on account of a cardiac or pulmonary exacerbation or frequent exacerbations treated at the outpatient clinic. The exclusion criteria were neurological, orthopedic, or peripheral vascular conditions preventing the patient from performing exercise; hemodynamic significant valvular disease; and proven cardiac ischemia or heart rhythm disturbances at a low-intensity exercise level. All patients provided written informed consent. The study was approved by the local medical ethical committee of Máxima Medical Center and was conducted in accordance with the declaration of Helsinki. The study was registered in the Netherlands Trial Register (NL9182).

### Outcome Measures

#### Program Completion and Adherence

The primary end points—program completion and adherence—were defined as the percentage of participants who completed the 8-week exercise program and the percentage of completed exercise sessions after receiving a prescription, respectively. To assess adherence, the participants were asked to record their exercise sessions with a wrist-worn activity tracker (Galaxy Watch Active; Samsung Electronics; see *Digital Platform and Activity Tracker* section). On the basis of the study population, characterized by a high disease severity and the presence of several comorbidities, we acknowledge that program completion and adherence are challenging. Nevertheless, given the fact that the entire program was highly personalized, performed in the patients’ home environment, and supported by fairly easy-to-use technology, we hypothesized that most of the participants would be able to complete the program and that adherence to the exercise prescription would be at least 70%.

#### Patient Satisfaction

Patient satisfaction and possible barriers to adherence were assessed with the validated Client Satisfaction Questionnaire-8 (CSQ-8) [15]. This self-administered questionnaire consists of 8 items, scored on 4-point Likert-type scales. For every patient, a total satisfaction score is calculated with a minimum score of 8 and a maximum score of 32, where higher scores indicate a higher level of satisfaction [16].

Moreover, a self-administered, Purpose-Designed Questionnaire (PDQ) was completed by each participant (Multimedia Appendix 1). This questionnaire consists of 26 items categorized into 5 topics: provision of information, contact with the therapists, safety, digital platform and activity tracker, and treatment results. Of the 26 items, 23 are scored on a 5-point Likert scale, resulting in a minimum score of 23 and a maximum score of 115 (a higher score indicates a higher level of satisfaction); 1 item is answered with yes or no; 1 item requires the participant to grade (from 0=very bad to 10=excellent) the program in general; and 1 item is an open question seeking comments or suggestions about the program.

#### Program Efficacy

Subjective program efficacy was evaluated through the following 3 questionnaires or rating scales, which were administered at baseline and again at the end of the program:

- Patient-Specific Complaints (PSC) Instrument. This tool asks the patient to mention 2-3 activities that are difficult to perform because of cardiopulmonary complaints. Each activity is then rated on a scale from 0 (very easy to perform the activity) to 10 (impossible to perform the activity). A mean PSC score is calculated by dividing the total PSC score by the number of chosen activities.
- Modified Medical Research Council (mMRC) Dyspnea Scale [17], which measures the degree of dyspnea severity during daily life activities. This self-administered tool rates the degree of disability in daily life caused by breathlessness on a scale from 0 to 4, where 0 represents not troubled by breathlessness except during strenuous exercise, and 4 represents too breathless to leave the house or breathless when dressing or undressing.
- Self-administered EuroQol 5-Dimension, 5-Level (EQ-5D-5L) Questionnaire, which measures HRQoL and which consists of 5 questions in 5 domains (mobility, self-care, usual activities, pain or discomfort, and anxiety or depression) [18]. Each question is answered on a 5-point severity scale. Moreover, the questionnaire comprises a visual analog scale (from 0=worst imagined health to 100=best imagined health) to grade HRQoL.

The objective measures to evaluate program efficacy, extracted at baseline and again at the end of the program were as follows:

- Number of repetitions during the 1-minute sit-to-stand (1-MSTS) test, which is a valid and responsive tool for measuring exercise capacity [19]. The patients were asked to sit on a chair, which was stabilized against the wall, with their arms crossed over their chest, their knees in an approximate 90-degree angle, and feet flat on the floor. They were asked to perform as many sit-to-stand cycles as possible in 1 minute. The patients were asked to fully stand up and then sit back again, touching the surface of the seat but not the backrest. They were not encouraged during the test but were informed when there were 10 seconds left, timed with a stopwatch.
- Handgrip strength as a measure of muscle strength, measured with the Jamar hydraulic hand dynamometer (JLW Instruments) [20]. The patients were asked to be seated, with shoulders in neutral position, elbows flexed at 90 degrees, and wrists in neutral position with the thumb facing upward. The forearm was not to rest on the arm of the chair. Three trials of handgrip strength were performed using each hand, beginning with the dominant hand. The patients were encouraged to squeeze as hard as they could. The maximum grip strength from all 6 trials was used.

### Exercise Program

The exercise program started with a combined intake supervised by both the physiotherapist and occupational therapist at the outpatient physiotherapy clinic of Máxima Medical Center. The intake consisted of an assessment of the daily life activities of each participant and the perceived barriers. On the basis of this interview, 2 to 3 problematic activities were defined that the participant wished to improve. These activities were rated with the PSC Instrument and were translated into patient-specific rehabilitation goals. In addition, the participants were instructed to complete the EQ-5D-5L Questionnaire and the mMRC Dyspnea Scale and to perform the 1-MSTS and handgrip tests.

The 8-week home-based exercise program consisted of a combination of endurance and strength exercise training tailored to the participants’ preferences in exercise modality (ie, walking, cycling, or swimming) and availability of training equipment at home. Exercises for (respiratory) muscle strengthening, breathing techniques, and techniques for mobilization of sputum were provided through instruction videos on the digital platform. The amount, duration, and content of the exercise sessions was determined individually by the physiotherapist. The intensity of the prescribed exercise sessions was guided by the rate of perceived exertion (Borg Rating of Perceived Exertion 6-20 Scale), which was filled in on the digital platform by the participant after a completed exercise session. The exercise sessions were recorded by the participant with a wrist-worn activity tracker (see *Digital Platform and Activity Tracker* section).

The occupational therapist primarily focused on the execution of daily life activities and energy balance. For this purpose, the digital platform was supplied with an activity diary to be filled in by the participant, which could be rated afterward by the occupational therapist.

The participants had a weekly video consultation through the digital platform with the physiotherapist or occupational therapist to discuss their progress and to adjust the training scheme if needed. However, the therapists had the option to adjust the frequency of the video consultations based on shared decision-making with the participant.

At the end of the program, an evaluation took place at the outpatient clinic, again with both therapists. The participants’ personal goals were evaluated through the PSC Instrument. In addition, the 1-MSTS and handgrip tests were administered again, along with the EQ-5D-5L Questionnaire and mMRC Dyspnea Scale. Finally, each participant answered 2 questionnaires (CSQ-8 and PDQ) on their satisfaction with the program, and the responses were analyzed after all participants completed the study.

**Figure 1:**
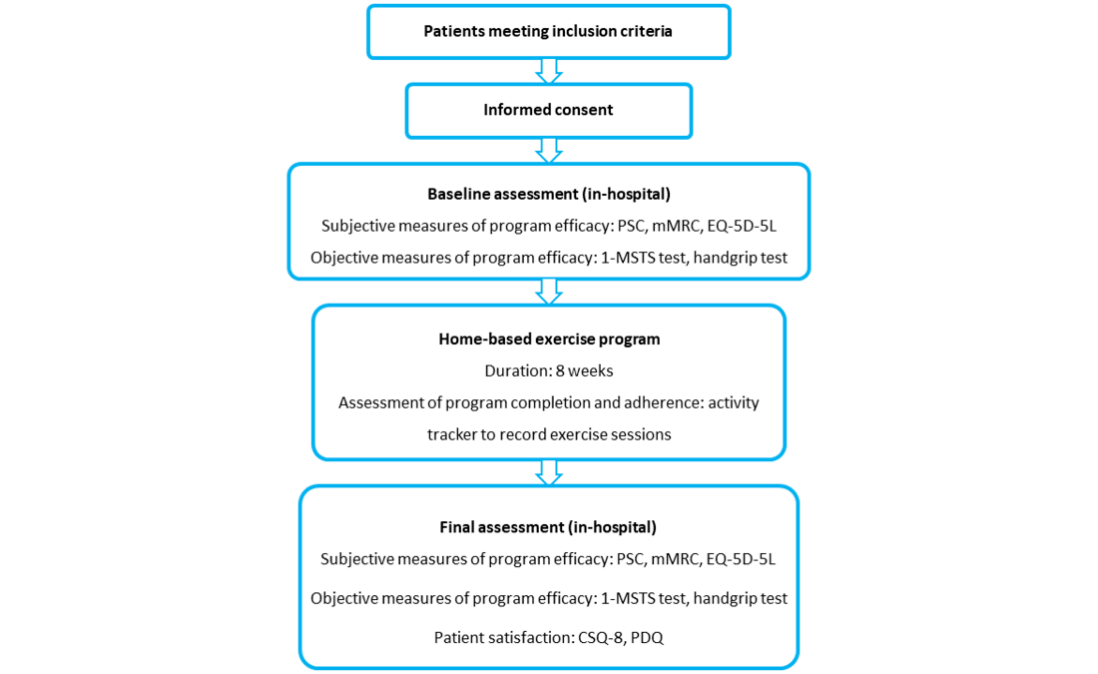
Participant timeline and study measurements. 1-MSTS: 1-minute sit-to-stand; CSQ-8: Client Satisfaction Questionnaire-8; EQ-5D-5L: EuroQol 5-Dimension, 5-Level Questionnaire; mMRC: modified Medical Research Council; PDQ: Purpose-Designed Questionnaire; PSC: Patient-Specific Complaints.

An overview of the participant timeline and study measurements is presented in Figure 1.

### Digital Platform and Activity Tracker

This study used a secure digital platform (Mibida BV). The eligible participants were already using this platform for telemonitoring of their cardiopulmonary disease. For the participants in this study, the platform was equipped with the following additional functionalities:

- An overview of the executed exercise sessions (recorded with the activity tracker). For each session, the following metrics were visualized: type of exercise, date of the exercise session, starting time of the session, duration of the session, heart rate course, and perceived Borg score. Actual feedback on these sessions was given by the therapists during the weekly videocall sessions.
- Short instruction films about exercises for (respiratory) muscle strengthening, breathing techniques, and techniques for mobilization of sputum. These films were recorded in advance in collaboration with the physiotherapy department. For each patient, a personalized set of films was selected by the physiotherapist and transferred to the digital platform.
- An activity diary for 1 or more days to be filled in by the participant, which could be scored afterward by the occupational therapist. These scores, represented as numbers, were discussed during the participants’ video consultations with the occupational therapist.
- A graphical overview of treatment goals based on the PSC list, with the associated PSC scores. The PSC scores could be filled in periodically by the participant (as was asked by the therapists) to evaluate progress.
- A videocall module for weekly communication with the therapists.
- A tab with reports created by the therapists. Tasks and assignments for the upcoming week were summarized here, as well as progress up to that point.

Exercise sessions were recorded with a wrist-worn activity tracker (Galaxy Watch Active). The watch was mainly used for evaluation of adherence, rather than for adjusting exercise prescription based on specific recorded metrics. The exercise recordings were automatically uploaded to the digital platform through the home Wi-Fi network of the patient.

### Statistical Analysis

For this study, we chose a convenience sample of 10 participants, rather than a calculation-based sample size. Nevertheless, we included a very homogeneous group of participants (older adults with a combined cardiopulmonary diagnosis and a recent exacerbation). For this reason, we considered a sample size of 10 patients sufficient to (1) explore whether the approach is feasible to execute on a larger scale and (2) obtain sufficient representative results to enable us to improve the intervention before upscaling. Descriptive statistics were used to describe the studied population with regard to the baseline characteristics and data on adherence, patient satisfaction, and program efficacy. Because of the small patient population, nonparametric tests were used. The Wilcoxon signed-rank test was used to compare differences in the PSC, mMRC, HRQoL, and 1-MSTS scores, as well as in handgrip strength, before and after the home-based exercise program, and the Friedman test was used to determine whether the change in adherence during the program was significant. Statistical analysis was performed using SPSS software (version 22; IBM Corp). For the analysis, the significance level was set at *P*<.05.

## Results

### Baseline Characteristics

The aim was to include 10 patients. Therefore, 13 patients were asked to participate, of whom 3 (23%) declined. Of these 3 patients, 2 (67%) provided reasons for declining to participate: (1) starting an extensive treatment for a comorbid disorder and not wanting to combine it with study participation and (2) feeling insecure about performing exercise that would not be directly supervised. The study participants were included between October 2019 and October 2020, had a median age of 71 (IQR 63-75) years, and 50% (5/10) were men. Additional patient baseline characteristics are presented in Table 1, and baseline cardiopulmonary patient profiles are presented in Table 2. Of the 10 participants, 9 (90%) were already familiar with using the digital platform for telemonitoring purposes—the median duration of the use of this platform was 20 (IQR 4-24) months—whereas 1 (10%) participant had never used the digital platform previously because telemonitoring had not started yet for this participant.

**Table 1:** Baseline characteristics (N=10).

Characteristics		Values
Age (years), median (IQR)		71 (63-75)
**Gender, n (%)**		
	Male	5 (50)
	Female	5 (50)
Height (m), median (IQR)		1.68 (1.60-1.84)
Weight (kg), median (IQR)		86 (71-96)
BMI (kg/m^2^), median (IQR)		28.2 (27.4-32.1)
Number of comorbidities^a^, median (IQR)		3.0 (0.8-4.3)
Cardiac and pulmonary exacerbations^b^, year before inclusion, median (IQR)		3.5 (1.8-6.0)
LTOT^c^, n (%)		6 (60)
**mMRC^d^** **Dyspnea Scale, n (%)**		
	0	0 (0)
	1	1 (10)
	2	1 (10)
	3	4 (40)
	4	4 (40)

^a^Chronic diseases other than cardiopulmonary diagnosis.

^b^Events characterized by worsening of respiratory symptoms and/or peripheral edema beyond normal day-to-day variation that led to change in medication (ie, diuretics, inhalers, systemic corticosteroids, antibiotics, or a combination). Events might be accompanied by a hospital admission.

^c^LTOT: long-term oxygen therapy.

^d^mMRC: modified Medical Research Council.

**Table 2:** Baseline cardiopulmonary patient profiles (N=10).

Patient number	Cardiac diagnosis	LVEF^a^ (%)	Pulmonary diagnosis	DLCO^b^
1	HFpEF^c^	63	COPD^d^ GOLD^e^ IV, group A	43
2	HFrEF^f^	35	COPD GOLD IV, group D	40
3	HFmrEF^g^	47	COPD GOLD II, group B	53
4	HFpEF	53	COPD GOLD IV, group D; recent COVID-19 infection	25
5	HFpEF	60	Asthma, bronchiectasis	—^h^
6	HFpEF	63	COPD GOLD II, group D	29
7	HFrEF	35	Asthma, unilateral emphysema	50
8	HFmrEF	40	COPD GOLD IV, group D	31
9	HFrEF	35	COPD GOLD II, group B; recent COVID-19 infection	68
10	HFpEF	65	COPD GOLD IV, group D	26

^a^LVEF: left ventricular ejection fraction.

^b^DLCO: diffusing capacity for carbon monoxide.

^c^HFpEF: heart failure with preserved ejection fraction.

^d^COPD: chronic obstructive pulmonary disease.

^e^GOLD: Global Initiative for Chronic Obstructive Lung Disease.

^f^HFrEF: heart failure with reduced ejection fraction.

^g^HFmrEF: heart failure with midrange ejection fraction.

^h^Not available.

### Study Completion and Adherence

**Figure 2:**
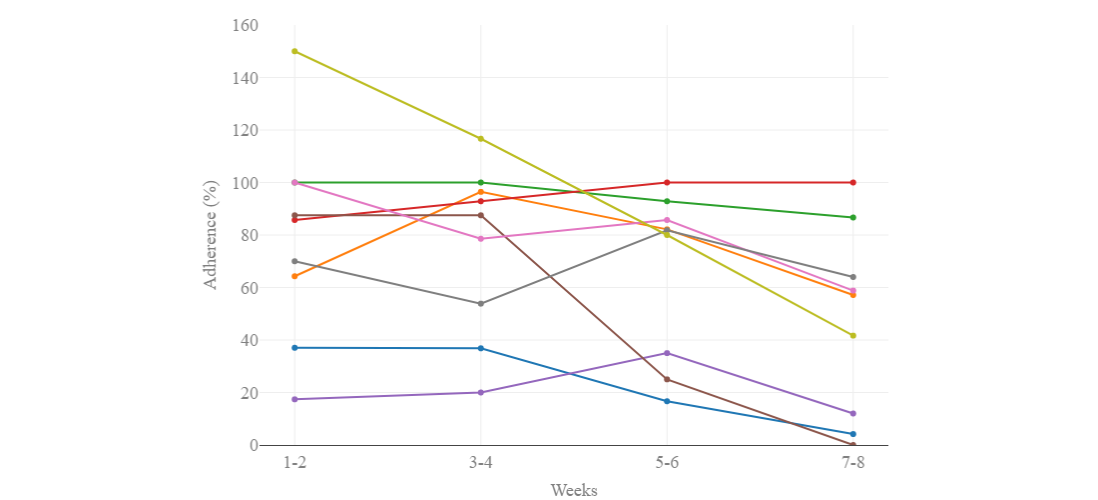
Interpersonal variability in adherence (n=9). Each colored line represents 1 participant.

Of the 10 participants, 9 (90%) completed the 8-week exercise program, whereas 1 (10%) participant ended the program prematurely because of a hospital admission (neither related to cardiac or pulmonary disease, nor to study participation) shortly after inclusion and inability to restart the program after discharge. The median adherence (ie, the percentage of completed exercise sessions after receiving the prescription) over 8 weeks was 75% (IQR 37%-88%), ranging from 20% to 95% (Figure 2).

**Figure 3:**
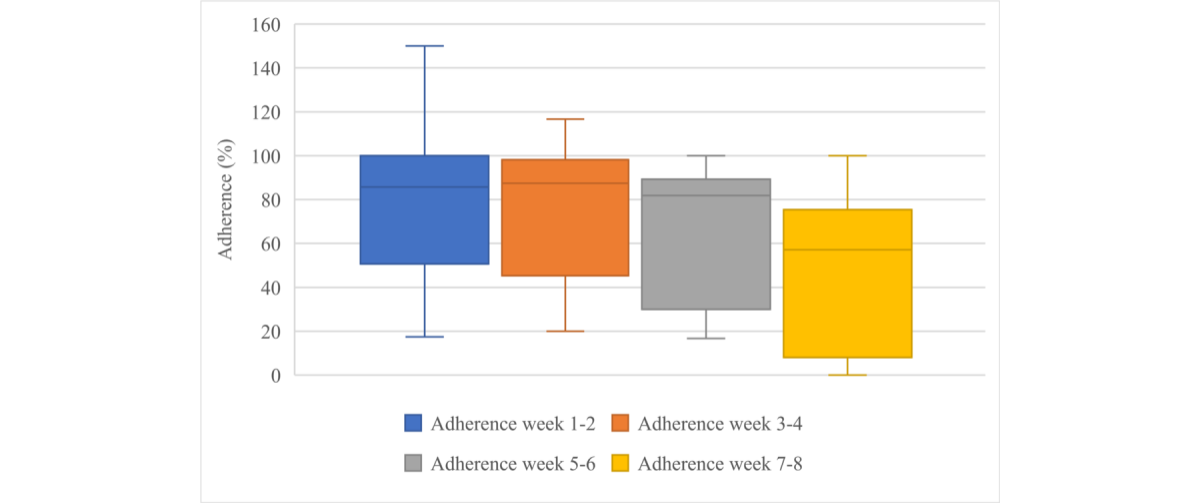
Boxplots showing median adherence over periods of 2 weeks (n=9).

When we divided the program into periods of 2 weeks, the median adherence decreased significantly: from 86% (IQR 51%-100%) in the first 2 weeks of the program to 57% (IQR 8%-75%) in the final 2 weeks of the program (*P*=.03; Figure 3).

### Adverse Events

During the study period, no adverse events occurred that were related to the home-based exercise program. Of the 9 participants who completed the program, in the case of 1 (11%) participant, the study was interrupted for several weeks by a severe pulmonary exacerbation in combination with a COVID-19 infection. This participant successfully restarted the study after recovery.

### Patient Satisfaction

#### CSQ-8 Score

**Figure 4:**
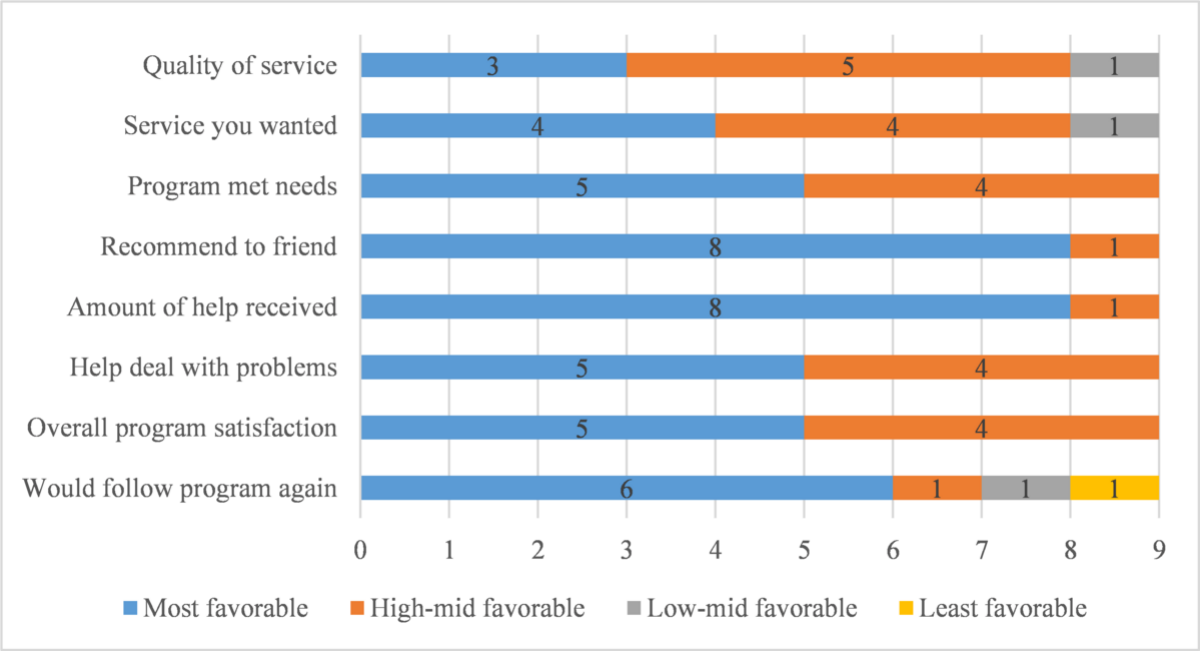
Distribution of each item of the Client Satisfaction Questionnaire-8 (n=9). Data are presented as the number of participants who provided a certain answer to each question.

The median total CSQ-8 score was 29 (IQR 26-32), which indicates a high level of satisfaction. The total CSQ-8 score ranged between a minimum of 21 and a maximum of 32. Figure 4 illustrates the distribution of each item of the CSQ-8, showing that CSQ questions 4 and 5 were scored best: 89% (8/9) of the participants were highly satisfied with the amount of help received and would definitely recommend the program to a friend, 67% (6/9) would definitely follow the program again, whereas of the 9 participants who completed the program, 1 (11%) participant would definitely not follow it again.

#### PDQ Score

The median total PDQ score was 103 (IQR 92-108), indicating a high level of satisfaction. The total PDQ score ranged between a minimum of 71 and a maximum of 113.

**Figure 5:**
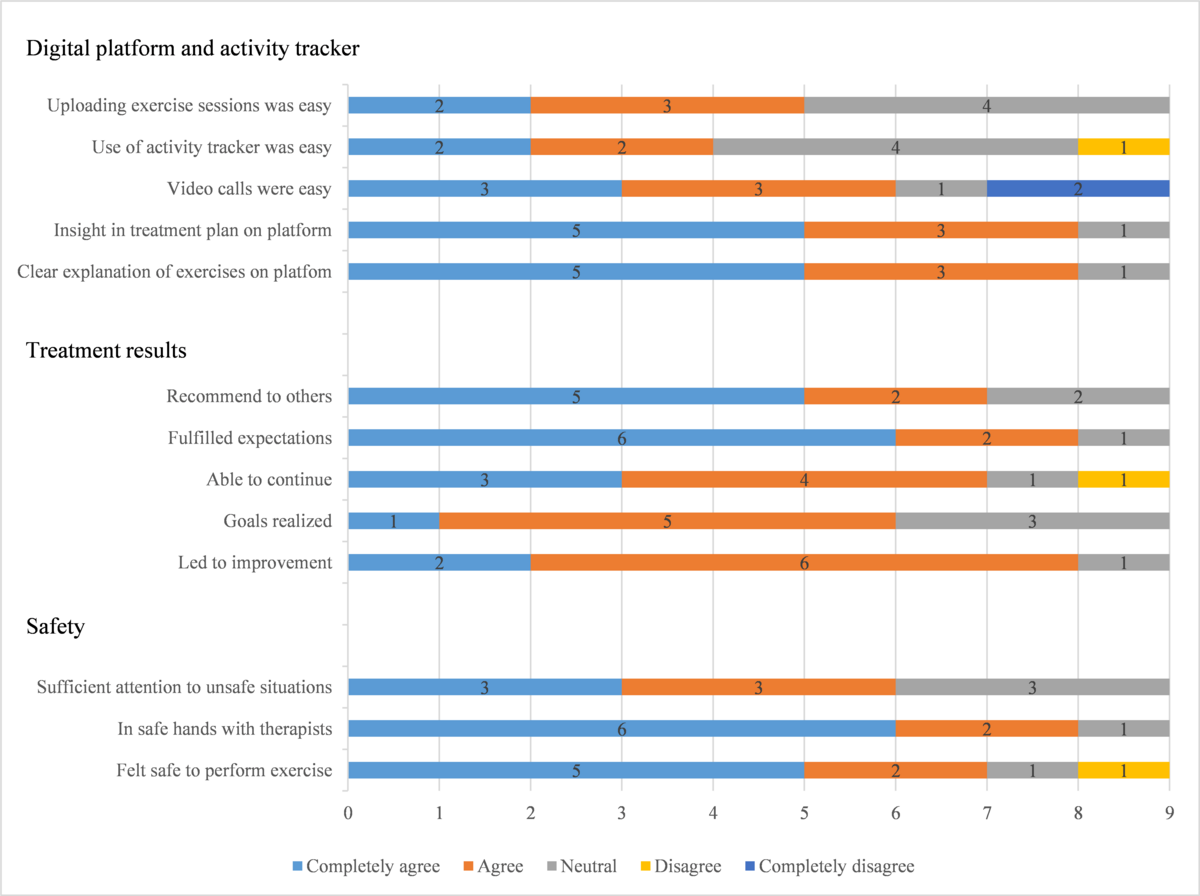
Distribution of each item in 3 topics of the Purpose-Designed Questionnaire (n=9). Data are presented as the number of participants who provided a certain answer to each question.

The subset of items in the topics provision of information (3 questions) and contact with the therapists (7 questions) were responded to with agree or completely agree by 6 to 9 of the participants, indicating high levels of satisfaction. No participant disagreed with these items. Figure 5 shows that the remainder of the topics (digital platform and activity tracker, treatment results, and safety) had a slightly broader distribution. Generally, agreement with the items of these topics was acceptable; however, regarding the digital platform and activity tracker, of the 9 participants who completed the program, 2 (22%) completely disagreed with the statement that making video calls was easy and 1 (11%) disagreed with the statement that the use of the activity tracker was easy, whereas for the topic treatment results, 1 (11%) disagreed with the statement about being able to continue the exercises after the conclusion of the program. Regarding safety, of the 9 participants, 7 (78%) completely agreed or agreed with the statement regarding whether they felt it was safe to perform the exercises at home, 1 (11%) chose the neutral response, and 1 (11%) disagreed.

The participants gave the program a median grade of 9.0 (IQR 8.0-9.5), where a grade of 0 represents very bad and 10 represents excellent. The final question of the PDQ is an open one, which gives participants the option to write down comments. Of the 9 participants, 6 (67%) answered this question. The most frequently reported comments were experiencing trouble making a proper video connection with the therapists (3/9, 33%) and experiencing trouble with recording the exercise sessions with the activity tracker (4/9, 44%). Of the 9 participants, 4 (44%) answered yes to the question regarding whether they experienced technical issues to a certain extent.

### Program Efficacy

#### Subjective Measures

The PSC score significantly decreased after completion of the program, from median 7.5 (IQR 6.1-8.9) to median 5.7 (IQR 3.8-6.7; *P*=.01). The mMRC dyspnea score decreased nonsignificantly after completion of the program, from median 3.0 (IQR 2.8-4.0) to median 2.0 (IQR 1-3; *P*=.07). The HRQoL score showed some trends toward improvement; however, no statistically significant changes were observed (Table 3).

**Table 3:** Health-related quality of life (measured with the self-administered EuroQol 5-Dimension, 5-Level Questionnaire) before and after the program (N=9).

		Baseline	After program completion	*P* value	
**Mobility, n (%)**					.32
	No problems	1 (11)	0 (0)		
	Slight problems	0 (0)	2 (22)		
	Moderate problems	5 (56)	6 (67)		
	Severe problems	3 (33)	1 (11)		
	Unable to walk	0 (0)	0 (0)		
**Self-care, n (%)**					.99
	No problems	4 (44)	4 (44)		
	Slight problems	3 (33)	2 (22)		
	Moderate problems	0 (0)	2 (22)		
	Severe problems	2 (22)	1 (11)		
	Unable to wash or dress	0 (0)	0 (0)		
**Usual activities, n (%)**					.16
	No problems	2 (22)	2 (22)		
	Slight problems	1 (11)	3 (33)		
	Moderate problems	2 (22)	3 (33)		
	Severe problems	4 (44)	1 (11.1)		
	Unable to do usual activities	0 (0)	0 (0)		
**Pain or discomfort, n (%)**					.05
	No pain or discomfort	1 (11)	4 (44)		
	Slight pain or discomfort	6 (67)	4 (44)		
	Moderate pain or discomfort	2 (22)	1 (11)		
	Severe pain or discomfort	0 (0)	0 (0)		
	Extreme pain or discomfort	0 (0)	0 (0)		
**Anxiety or depression, n (%)**					.56
	Not anxious or depressed	5 (56)	7 (78)		
	Slightly anxious or depressed	3 (33)	0 (0)		
	Moderately anxious or depressed	1 (11)	2 (22)		
	Severely anxious or depressed	0 (0)	0 (0)		
	Extremely anxious or depressed	0 (0)	0 (0)		
VAS^a^, median (IQR)		50.0 (42.5-70.0)	60.0 (50.0-75.0)	.09	

^a^VAS: visual analog scale.

#### Objective Measures

The number of repetitions during the 1-MSTS test showed a nonsignificant increase after completion of the program, from a median of 15 (IQR 12-19) repetitions to a median of 16 (IQR 13-21) repetitions (*P*=.05). In addition, no significant differences in handgrip strength were observed, from a median of 30 (IQR 25-34) kg to a median of 30 (IQR 23-33) kg (*P*=.73).

### Financial Costs

The financial costs of the program can be divided into (1) costs with regard to the deployment of health care professionals and (2) costs with regard to technology (activity tracker, digital platform, and laptops that were needed for the therapists to perform video consultations). An overview of the intervention costs per participant is presented in Table 4.

**Table 4:** Intervention costs per participant.

Type of costs		Costs per participant € (US $)
**Health care professionals**		
	Intake with 2 therapists (1 hour)	79.94 (92.69)
	Video consultations (6×15 minutes)	59.96 (69.53)
	Final evaluation with 2 therapists (30 minutes)	39.97 (46.35)
Activity trackers (2 months)		8.33 (9.66)
Hosting costs of digital platform (2 months)^a^		13 (15)
Laptops (2 months)^a^		0.31 (0.36)
Total costs per participant per program		201.51 (233.66)

^a^Based on an annual number of 200 participants in regular care setting.

## Discussion

### Overview

This exploratory study showed that home-based cardiopulmonary telerehabilitation for patients with severe combined cardiac and pulmonary disease is feasible in terms of program completion and is associated with high satisfaction rates and functional improvement. However, a decrease in adherence during the program was observed, and some participants reported difficulties with the technology.

### Program Completion and Adherence

Our study showed that of the 10 participants, 9 (90%) completed the program, but the median adherence was lower (75%; (IQR 37%-88%). It should be noted, however, that the actual adherence may have been underestimated because we based our findings solely on the number of recorded sessions with the activity tracker, which was experienced as troublesome by some patients. Other studies evaluating home-based CR in patients with CHF generally showed a high mean adherence but with a wide variation ranging from 54% to 110% [21]. In patients with COPD too, adherence to home-based exercise programs was highly variable, ranging from 21% [22] to 93.5% [23]. The study by Bernocchi et al [14] reported both a high completion rate of 86% and high adherence of 93% during a 4-month home-based rehabilitation program for patients with middle-severe combined CHF and COPD. However, because adherence was monitored by means of a written physical activity diary compared with sensor-based registration in our study, it is difficult to directly compare these results. Other factors explaining the broad variation in the adherence rates in home-based programs include the design of the program (eg, directly supervised home-based sessions vs unsupervised sessions); the number of contact moments with the telerehabilitation institution; the use of motivational strategies during the program; and the population being studied with regard to age and frailty, disease severity, clinical stability of the disease, and presence of comorbidities.

Although completion of the telerehabilitation program was high, we observed a decrease in adherence to the prescribed exercise sessions during the study period. A possible explanation for reduced adherence is temporary worsening of cardiopulmonary physical complaints or other coexisting diseases. Although the patients were also participating in a telemonitoring program focused on symptoms and vital signs, an actual integration of telemonitoring and the home-based exercise program was lacking. Ideally, when worsening of physical complaints was detected by telemonitoring, the rehabilitation therapists should have been informed immediately to adjust the exercise program to the patients’ physical capacity. Indeed, the study by Loeckx et al [24] showed that a lack in program adjustment during an exacerbation of COPD was experienced as a barrier by patients with COPD attending a 12-week telecoaching program. Furthermore, the decline in adherence may be related to motivational problems. This is supported by the fact that the median adherence to exercise prescription in our study gradually decreased (as opposed to a sudden decrease because of symptoms). This gradual decrease in adherence is not unique to our study and was also found in other studies on home-based exercise programs in both CHF and COPD [25-27]. This highlights one of the most challenging aspects of CR and PR programs, which is to achieve actual behavioral changes that result in long-term maintenance of a certain activity level after completion of a rehabilitation program. This might be even more challenging in older patients with several comorbidities who have frequent relapses of their disease. Home-based rehabilitation programs should therefore be designed using effective behavior change techniques. Our study used the following behavior change techniques: goal setting, action planning, using graded tasks, self-monitoring of behavior, motivational interviewing during follow-up video calls, information about health consequences, and reviewing outcome goals. The number of techniques used is in line with other studies on eHealth physical activity interventions in patients with cardiovascular disease [28]. Behavior change techniques that might have been of added value in this study are the use of social support, providing prompts or cues to perform exercise, and providing rewards when an exercise session was completed (eg, by using text messages). Furthermore, a step-down approach to contact moments with the therapists and on-demand coaching may be more effective than a fixed coaching frequency and period. Nevertheless, the effectiveness of individual behavior change techniques with regard to physical activity outcome is not established yet [28].

Although digital technology can serve as a very useful medium to apply behavior change techniques, it should be noted that some of our study participants experienced difficulties using digital technology. Therefore, the technology can also serve as a barrier to achieving behavioral changes. In fact, Tadas et al [29] found that although the use of technology in eHealth interventions for patients with cardiovascular disease is readily accepted, the use of additional devices, especially in older adults, can be perceived as burdensome. Nevertheless, the facilitators with regard to the adoption of digital technology were background knowledge about the disease, in-the-moment understanding of health status by monitoring of physical (activity) data, the ability to connect and exercise with other patients and the involvement of family, receiving direct feedback, reminders through apps that might also use gamification principles, and the ability to personalize the program in terms of technical aspects [29]. The adoption of digital health technology in patients with COPD is associated with similar barriers and facilitators [30].

### Program Completion and the COVID-19 Pandemic

The COVID-19 pandemic has added new barriers to participation in center-based CR and PR, namely temporary closure of CR and PR centers, reduced capacity after partial reopening of these centers, and anxiety of patients regarding regular visits to a CR or PR center [31]. This study was predominantly performed during the COVID-19 pandemic, but the inclusion of participants and follow-up consultations by the therapists were not affected by the pandemic. Moreover, adherence to exercise prescription is likely to be unaffected by the pandemic because exercise could be performed in the home environment, taking the patients’ preferences into account. This study thereby shows the potential of home-based interventions as an alternative to center-based rehabilitation.

### Program Satisfaction

Although the participants in our study were highly satisfied with the home-based exercise program, they were experiencing difficulties in particular with using the wrist-worn activity tracker, which was used to record their exercise sessions. To facilitate the activity tracker’s use, one of its software features was adjusted before the study to enable the participants to start and end training sessions more easily. Moreover, functionalities that were not needed for study purposes were turned off. Nevertheless, some participants still experienced the use of the activity tracker as troublesome, which may have contributed to impaired adherence to the exercise prescription. Therefore, we suggest the use of sensors that patients only need to wear, without the need to perform additional actions. Moreover, automatic transfer of sensor data to a health platform should be provided. In addition, sensors that provide simple metrics (such as step count) and give direct feedback to users have been particularly graded as useful [24].

### Program Efficacy

Program efficacy was assessed using both subjective and objective measures. Although the sample size was too small to evaluate program efficacy with sufficient statistical power, and a control group was lacking, we observed an improvement in the PSC scores. Moreover, a trend toward improvement in the mMRC Dyspnea Scale and HRQoL scores was observed. No clinically relevant changes were observed in the objective measures (1-MSTS and handgrip strength tests). Although several studies on home-based CR and PR showed evidence of improvement in QoL and physical performance, as well as reduction in hospital admissions [32], this cannot yet be confirmed in our studied patient population, which is characterized by severe combined cardiopulmonary disease in combination with several other comorbidities and frequent exacerbations. This is supported by the study by Demeyer et al [33], which showed a significant improvement in physical activity level in patients with COPD across the whole spectrum during a telecoaching intervention but demonstrated attenuated effects in patients with a higher disease severity.

### Future Research

This exploratory study aims to optimize the intervention for a larger trial that will assess the effectiveness of home-based rehabilitation in patients with severe combined cardiac and pulmonary disease. On the basis of the findings of this study, we suggest making the following adjustments to optimize the intervention:

- Integration of telemonitoring strategies and telerehabilitation, that is, creating the ability to adjust exercise prescription in line with fluctuations in symptoms and exercise tolerance.
- Using prompts to stimulate participants to perform their exercise and also offering rewards after completing an exercise session, for example, through text messages.
- Use of a sensor that is easy to use, does not need the participant to perform several additional actions, provides metrics that are easy to interpret, and offers direct feedback.
- A treatment period of 8 weeks was experienced as rather short by the therapists with regard to providing sufficient coaching and education for the participants to achieve their rehabilitation goals. Although a duration of 8 weeks is more often used in rehabilitation programs, a prolonged period of coaching (at least 12 weeks) in this population consisting of patients with complex health conditions might result in higher program efficacy. A step-down approach to contact moments with the therapists might be of added value to be able to extend the program duration without causing an additional financial burden. Moreover, it might stimulate participants to perform their exercise without frequent feedback from the therapists, thereby preparing them for the period after the rehabilitation program has ended.

### Limitations

This study included a few limitations. First, the participants were already using the digital platform for telemonitoring purposes. The familiarity with the platform might have resulted in higher satisfaction rates with the home-based exercise program. Of the 10 participants, 1 (10%) was not familiar with the platform before beginning the program; however, this participant also scored high on satisfaction. Moreover, in a future study, home-based telerehabilitation will also be introduced after a period of telemonitoring because of the instability of the diseases.

Second, our study only focused on physical activity and not on other lifestyle domains. However, the ability to improve and maintain a certain activity level will also be influenced by other lifestyle behavior domains such as dietary behavior, mental stress, emotional well-being, and sleep quality. These factors were not taken into account in this rehabilitation program.

Finally, the results of this trial should be interpreted with caution because of the small number of participants and the lack of a control group. Nevertheless, because this study aims to explore program completion and adherence to, as well as patient satisfaction with, a home-based exercise program to facilitate the design of a future randomized controlled trial, we chose not to include a control group.

### Conclusions

Home-based telerehabilitation in older patients with combined severe cardiopulmonary disease and multiple other comorbidities is feasible in terms of a high program completion rate and high levels of patient satisfaction. However, a trend toward decline in adherence was observed during the program, which highlights the need for adding technology-based behavior change techniques in future trials.

## Appendix

Multimedia Appendix 1 An example of the Purpose-Designed Questionnaire.

## Abbreviations

1-MSTS: 1-minute sit-to-stand
CHF: chronic heart failure
COPD: chronic obstructive pulmonary disease
CR: cardiac rehabilitation
CSQ: Client Satisfaction Questionnaire
EQ-5D-5L: EuroQol 5-Dimension, 5-Level
HRQoL: health-related quality of life
mMRC: modified Medical Research Council
PDQ: Purpose-Designed Questionnaire
PR: pulmonary rehabilitation
PSC: Patient-Specific Complaints
