# Supplementary material for: Home-Based Exercise Program for Patients With Combined Advanced Chronic Cardiac and Pulmonary Diseases: Exploratory Study
Source: JMIR Form Res. 2021 Nov 9;5(11):e28634. doi: 10.2196/28634 (PMC8663616; doi:10.2196/28634)
Supplement: Multimedia Appendix 1 [file formative_v5i11e28634_app1.docx]

**Multimedia Appendix 1. An example of the Purpose-Designed Questionnaire**

*Provision of information*

1. I received sufficient and clear information about the treatment.

completely disagree/disagree/neutral/agree/completely agree

1. Before the treatment started, the therapists clearly explained, what they were going to do.

completely disagree/disagree/neutral/agree/completely agree

1. I got the feeling that I was able to object to the treatment if wanted to do this.

completely disagree/disagree/neutral/agree/completely agree

*Contact with the therapists*

1. I was treated with respect and kindness by the therapists.

completely disagree/disagree/neutral/agree/completely agree

1. I felt comfortable during the treatment.

completely disagree/disagree/neutral/agree/completely agree

1. I have had enough possibilities to ask questions.

completely disagree/disagree/neutral/agree/completely agree

1. I had the feeling that the therapists listened to me.

completely disagree/disagree/neutral/agree/completely agree

1. My therapists worked together in a good way.

completely disagree/disagree/neutral/agree/completely agree

1. I received treatment by the same therapists.

completely disagree/disagree/neutral/agree/completely agree

1. My therapists had enough time for me.

completely disagree/disagree/neutral/agree/completely agree

*Safety*

1. I felt save to perform exercise at home/without supervision.

completely disagree/disagree/neutral/agree/completely agree

1. I felt in safe hands with my therapists.

completely disagree/disagree/neutral/agree/completely agree

1. My therapists paid sufficient attention to unsafe situations.

completely disagree/disagree/neutral/agree/completely agree

*Use of digital platform and activity tracker*

1. The explanation of different exercises on the digital platform was clear to me.

completely disagree/disagree/neutral/agree/completely agree

1. I had insight in my treatment plan on the digital platform.

completely disagree/disagree/neutral/agree/completely agree

1. Making video calls with the therapists was easy.

completely disagree/disagree/neutral/agree/completely agree

1. The use of the activity tracker was easy.

completely disagree/disagree/neutral/agree/completely agree

1. Uploading exercise sessions was easy.

completely disagree/disagree/neutral/agree/completely agree

1. Did you miss exercise sessions because of technical issues?

yes/no

*Treatment results*

1. The treatment has lead to improvement of my health status or reduction of my physical complaints.

completely disagree/disagree/neutral/agree/completely agree

1. My treatment goals are realized.

completely disagree/disagree/neutral/agree/completely agree

1. The treatment fulfilled my expectations.

completely disagree/disagree/neutral/agree/completely agree

1. I think I can continue with exercising by myself.

completely disagree/disagree/neutral/agree/completely agree

1. I would recommend the therapy to others.

completely disagree/disagree/neutral/agree/completely agree

1. How would you grade the program? 0 means ‘very bad’, 10 means ‘excellent’.

*Suggestions or comments*

Do you have any suggestions for improvement of the current treatment? Other comments can also be written down here.
